# Gene expression profiles in developing nephrons using *Lim1 *metanephric mesenchyme-specific conditional mutant mice

**DOI:** 10.1186/1471-2369-7-1

**Published:** 2006-02-07

**Authors:** You-Tzung Chen, Akio Kobayashi, Kin Ming Kwan, Randy L Johnson, Richard R Behringer

**Affiliations:** 1 Department of Molecular Genetics, University of Texas M. D. Anderson Cancer Center, Houston, Texas 77030, USA; 2 Department of Biochemistry and Molecular Biology, University of Texas M. D. Anderson Cancer Center, Houston, Texas 77030, USA

## Abstract

**Background:**

*Lim1 *is a homeobox gene that is essential for nephrogenesis. During metanephric kidney development, *Lim1 *is expressed in the nephric duct, ureteric buds, and the induced metanephric mesenchyme. Conditional ablation of *Lim1 *in the metanephric mesenchyme blocks the formation of nephrons at the nephric vesicle stage, leading to the production of small, non-functional kidneys that lack nephrons.

**Methods:**

In the present study, we used Affymetrix probe arrays to screen for nephron-specific genes by comparing the expression profiles of control and *Lim1 *conditional mutant kidneys. Kidneys from two developmental stages, embryonic day 14.5 (E14.5) and 18.5 (E18.5), were examined.

**Results:**

Comparison of E18.5 kidney expression profiles generated a list of 465 nephron-specific gene candidates that showed a more than 2-fold increase in their expression level in control kidney versus the *Lim1 *conditional mutant kidney. Computational analysis confirmed that this screen enriched for kidney-specific genes. Furthermore, at least twenty-eight of the top fifty (56%) candidates (or their vertebrate orthologs) were previously reported to have a nephron-specific expression pattern. Our analysis of E14.5 expression data yielded 41 candidate genes that are up-regulated in the control kidneys compared to the conditional mutants. Three of them are related to the Notch signaling pathway that is known to be important in cell fate determination and nephron patterning.

**Conclusion:**

Therefore, we demonstrate that *Lim1 *conditional mutant kidneys serve as a novel tissue source for comprehensive expression studies and provide a means to identify nephron-specific genes.

## Background

Kidney is one of the main excretory and homeostatic organs of the body. The basic structural and functional unit of the kidney is the nephron. The development of a nephron involves a series of reciprocal tissue inductions between the ureteric bud and the metanephric mesenchyme. Induced metanephric mesenchyme cells condense to form pretubular cell aggregates and go through a mesenchyme to epithelial transition and a series of morphological changes, including the formation of nephric vesicles, comma- and S-shaped bodies and eventually the formation of mature nephrons. A mature nephron is composed of the vascular loop of the glomerulus, Bowman's capsule, the proximal convoluted tubule, the loop of Henle and the distal convoluted tubule that connects to the drainage system. Genes expressed in developing and mature nephrons may be important for their development, structural integrity, and physiological function. In humans, mutations in such genes may cause kidney disease [1].

Mouse has been widely used as a model organism for biomedical research. This is because the mouse is anatomically and physiologically similar to human. Recent progress in the human and mouse genome projects further indicates that the organization of these two mammalian genomes are highly conserved [2]. Over 95% of human genes can find their counterparts in the mouse genome [3,4]. This high similarity between mouse and human underscore the use of the mouse as the model organism *par excellence *for studies of many aspects of human biology.

Although genes involved in kidney organogenesis or associated with kidney disease have been identified, there is still limited molecular genetic knowledge of kidney development and homeostasis. Recent progress in microarray technology provides a powerful tool to study the kidney [5-14]. Mice with mutations that alter specific aspects of kidney development and function provide unique tissue resources for microarray studies [15-18].

*Lim1*, also called *Lhx1*, is a LIM-class homeobox gene that is expressed in the ureteric bud and pretubular cell aggregate prior to epithelialization of the developing metanephric kidney [19,20]. Most *Lim1 *null mutants die around E10.5, an embryonic stage prior to the development of the metanephros [21]. Rare *Lim1*-null mutant mice survive to birth but do not have kidneys, demonstrating an essential role for this gene in kidney organogenesis [21]. To bypass the early lethality that hinders the analysis of *Lim1 *function in kidney organogenesis, a *Lim1 *conditional null allele in mouse was generated [22]. An *Rarb2-Cre *transgene was generated and used for metanephric mesenchyme-specific ablation of *Lim1 *that resulted in newborn mice that had kidneys but no nephrons [20].

Nephrogenesis is a continuous process that begins with the induction of metanephric mesenchyme by the ureteric bud, around embryonic day 10.5 (E10.5), and persists several weeks after birth in mice [1]. The first mature nephron is observed at E16.5 [23]. Histological analysis suggests that the development of *Lim1 *mutant nephrons stops at the nephric vesicle stage, which begins around E11.0. Loss of a nephric vesicle polarity marker, *Brn1*, expression in the E13.5 conditional mutant kidneys further indicated that the *Lim1 *is required for correct patterning of the nephric vesicle [20]. The developing nephric vesicle represents an important developmental stage in which nephron polarity is established. Disruption of its patterning results in a failure to form nephron structures such as proximal tubules and glomerular epithelium [24].

In this study, we hypothesized that *Lim1 *mutant nephron-deficient kidneys could be used as a novel tissue resource for microarray experiments to identify genes expressed in the developing nephrons. Kidneys of two developmental stages were examined. Control and conditional mutant kidneys of E14.5 mouse embryos were used to identify genes involved in early nephron development including pattern formation. In contrast, E18.5 kidneys were used to isolate functional genes that are expressed in mature nephrons.

## Methods

### Generation of conditional mutant mice and genotyping

All procedures performed on animals were done in accordance with guidelines of the American Physiological Society and were approved by The University of Texas MD Anderson Cancer Center Institutional Animal Care and Use Committee (Richard R. Behringer, IACUC Protocol Number: 02-90-01735). Mice carrying a targeted *Lim1 *null allele (*Lim1*^*lacZ*^, [25]), a *Lim1 *conditional null allele (*Lim1*^*flox*^, [22]) and an *Rarb2-Cre *transgene in which Cre is expressed in the metanephric mesenchyme of the developing kidney [20], were used in this study. *Lim1*^*lacZ*/+ ^and *Lim1*^*flox*/*flox *^mice were maintained on a C57BL/6J × 129/SvEv genetic background. *Rarb2-Cre *transgenic mice were initially generated on a C57BL/6J × SJL/J genetic background.

To obtain mouse embryos with metanephric mesenchyme-specific *Lim1 *deficient (*Lim1*^*flox*/*lacZ*^; *Rarb2-Cre*^*tg*/+^) kidneys, timed matings between *Lim1*^+/*lacZ*^; *Rarb2-Cre*^*tg*/+ ^males and *Lim1*^*flox*/*flox *^females were established. Kidney samples of two different embryonic stages (E14.5 and E18.5) were isolated. Their genotypes were assigned unambiguously using real time PCR assays detecting the presence of *lacZ *and *Cre *alleles, which were established in the M. D. Anderson Cancer Center DNA Analysis Core Facility. For the E14.5 time point, a total of 71 embryos were collected, 17 of them were genotyped as conditional mutants (*Lim1*^*flox*/*lacZ*^; *Rarb2-Cre*^*tg*/+^). Kidneys from 23 *Lim1*^*flox*/+^; *Rarb2Cre*^*tg*/+ ^embryos were used as "control" kidneys. For the E18.5 time point, a total of 39 embryos were harvested, 9 of them were genotyped as conditional mutants and 14 of them were genotyped as controls.

### Tissue collection and RNA preparation

Embryonic kidney tissue from each individual was placed in a separate tube with 0.5 ml TRIzol (Invitrogen, Carlsbad, CA) and stored at -75°C until the corresponding visceral tissue could be genotyped. After a genotype was unambiguously assigned to each individual, TRIzol preserved kidneys of the same genotype were pooled and total RNA was prepared as per the manufacturer's instructions. Total RNA was then processed using a QIAGEN RNeasy Midi Kit before *in vitro *transcription-labeling reaction per Affymetrix (Santa Clara, CA) recommendation. Once purified, RNA quality was determined by electrophoretic methods using an agarose gel or analysis using an Agilent Bioanalyzer 2100 (Palo Alto, CA) and by spectroscopy at 260 and 280 nm.

### Microarray processing

Five to forty micrograms of total RNA from each pooled embryonic kidney sample was used to produce the cRNA target for the microarray. The target was created using a reverse transcription reaction to produce cDNA (Supercript Choice System, Gibco), which was subsequently subjected to *in vitro *transcription with biotinylated cytidine-5'-triphosphate and uridine-5'-triphosphate using the ENZO BioArray High Yield RNA Transcript Labeling Kit to produce biotinylated cRNA. The target was then fragmented and hybridized to Mouse Genome 430 2.0 Affymetrix GeneChip Arrays (Affymetrix, Santa Clara, CA) in duplicates using an Affymetrix GeneChip Fluidics Station 400, according to the manufacturer's standard protocols. The arrays were stained with phycoerythrin-coupled avidin and scanned using a GeneArray Scanner 3000. The resultant output was analyzed using Affymetrix Microarray Suite software and examined for excessive background or evidence of RNA degradation. All microarray processing was performed in the Murine Microarray and Affymetrix Facility at the University of Texas M. D. Anderson Cancer Center.

After scanning, all probe sets were scaled to a signal intensity of 250 and relative levels of expression of each transcript (signal) were determined using Microarray Suite 5.0 software (Affymetrix). The images of all arrays were inspected for physical anomalies and for the presence of excessive background hybridization. Generally, all array results used in this study were of good quality, and no major manufacturer's defects or abnormalities were detected.

### Microarray analysis

Microarray experiment on each time point and genotype was performed in technical duplicates (ie. a single RNA preparation of pooled kidneys of one genotype used for two separate target preparations). Data from a total of 8 independent arrays were used in this study, 2 arrays were used for RNA samples from E18.5 control kidneys, 2 for E18.5 *Lim1 *conditional mutant kidneys, 2 for E14.5 control kidneys, and the other 2 for E14.5 *Lim1 *conditional mutant kidneys. Data generated from all arrays that satisfied the preliminary analysis were exported and loaded into DNA-Chip Analyzer (dChip2004) [26,27], where statistical and comparative analyses were performed to verify the data. The data were normalized using the default normalization method. Briefly, an iterative procedure was used to identify an invariant set of probes, which presumably consisted of non-differentially expressed genes. A piecewise-linear running median curve was then calculated and used as the normalization curve. After normalization, all arrays had similar brightness. Median intensities around 155 (between 155 to 158) were obtained after normalization. Percent gene present (P call%) values between 55.7% and 63.6% were observed using default detection p-value cut offs (a_1 _= 0.04 and a_2 _= 0.06). Array outlier (%) and single outlier (%) were detected at ranges from 0.016% to 0.080% and from 0.009% to 0.045%. Expression data obtained from all arrays used is provided in Additional file 1.

Normalized data were exported in a tab delimited text format. Fold changes of each transcript from different samples were calculated and sorted using Microsoft Excel 5.0 software. Signal obtained from control kidney samples were used as an experiment to compare to the signal obtained from *Lim1 *conditional mutant kidneys that was designated as a baseline. A 2-fold change in the means of signal obtained from experimental duplicates and those from baseline duplicates was used as the criterion to identify differentially expressed transcripts. To ensure the quality of the data, probe sets that showed a fold change between duplicates greater than between the experimental mean and baseline mean were removed. To study only genes that showed consistent expression on experimental chips, probe sets that did not show consistent present calls in the experimental duplicates were removed. To focus on genes with a significant fold change between the experiment and the baseline, only probe sets that the product of their experimental mean and fold change were more than 100 were retained. To produce a compact differentially expressed gene list, the probe set list was sorted within Microsoft Excel based on Locus Link number and redundant entries were removed. Our experimental design description and the data format provided in the Additional files fulfill the MIAME (minimum information about a microarray experiment) standards [28].

### Expression specificity and ontological analysis

To evaluate kidney expression specificity of identified genes, gene symbols and locus numbers were used to retrieve their relevant expression information in the Genomics Institute of the Novartis Foundation (GNF) Gene Expression Atlas 2 and Unigene databases. The GNF Atlas 2.0 contains two replicates each of 61 mouse tissues run over Affymetrix probe arrays. It was accessed using the Gene Sorter server provided by the University of California at Santa Cruz [29]. Gene Sorter provides a score, between -4 to 4, to describe the relative expression level of a gene in different tissues presented in the GNF Atlas 2 [30]. In contrast Unigene is a system automatically partitioning GenBank sequences, including expressed sequence tags (ESTs), into a non-redundant set of gene-oriented clusters [31]. The Unigene data were obtained from SOURCE [32], which provides a normalized expression level, based on the number of ESTs within the cluster found in cDNA libraries of different sources, expressed in percentages, to represent the relative abundance of a transcript in different tissues or organs [33]. To understand the composition of the genes identified in our study, ontological analysis was performed using DAVID (Database for Annotation, Visualization and Integrated Discovery) and EASE (Expression Analysis Systematic Explorer) from the National Institute for Allergy and Infectious Disease (NIAID) [34-37]. Data obtained were processed and charts were drawn using Microsoft Excel 5.0 software.

## Results

### Identification of stage-specific kidney genes

The first protocol used was to compare gene expression levels in kidney samples of the two developmental stages so that stage-specific kidney genes could be identified. To enrich for E18.5 kidney-specific genes, signals obtained from E18.5 control kidney samples (E18.5C) were used to compare with E14.5 control kidney data (E14.5C). Genes sorted according to their expression signal fold changes (E18.5C/E14.5C) generated a list enchriched for E18.5 mouse kidney genes. A list of 1,006 genes showed more than 2 fold change was identified (Table 1 and Additional file 2). As shown in Table 2, the enrichment for kidney specificity was dramatic. The average relative kidney expression level among the top 50 genes on the list reported by Gene Sorter is 3.6 whereas that of the gene list sorted using raw signals (E18.5C) is only -0.1. Similarly the average normalized expression level in the kidney, reported by SOURCE [33], of the top 50 genes also showed a nearly 15 fold increase (30.69% compare to 2.07%). Although a PubMed search did not find any of the top 50 genes in the list sorted by raw signals (E18.5C; 0%), 22 of the top 50 (44%) genes sorted by fold change (E18.5C/E14.5C) were described to have a nephron-specific expression pattern.

**Table 1:** Numbers of genes that showed more than a 2-fold increase in different expression level comparisons.

Experiment/Baseline	E18.5 C	E18.5 M	E14.5 C	E14.5 M
E18.5 C	-	47	796	-
E18.5 M	465	-	481	341
E14.5 C	1,006	476	-	2
E14.5 M	-	331	41	-

**Table 2:** Expression profile comparison between E18.5 control and *Lim1 *conditional mutant kidney generated a gene list enriched for nephron-specificity.

Sorted by	Relative kidney expression level (GNF 2.0)	Normalized expression in kidney (SOURCE)	Published Nephron-specific expression (PubMed)
E18.5 C	-0.1	2.07%	0%
E18.5 C/E14.5 C	3.6	30.69%	44%
E18.5 C/E18.5 M	3.5	32.27%	56%

E14.5 C	-0.1	1.48%	0%
E14.5 C/E18.5 C	0.0	2.51%	4%
E14.5 C/E14.5 M	2.9	21.51%	26%

The same approach was applied to the E14.5 experiment to identify 796 gene that showed more than a 2 fold change (Table 1 and Additional file 3), however the enrichment for kidney- or nephron-specificity was not significant. As shown in Table 2, the average relative kidney expression levels of the top 50 genes were not much different in lists sorted by the raw signals and fold changes (-0.1 and 0.0). A slight increase in the average normalized expression in the kidney was observed (1.48% and 2.51%). There were only 2 genes (4%) among the top 50 reported to have a nephron-specific expression pattern in the gene list sorted by developmental stage-specific fold change (E14.5C/E18.5C). A closer look of the gene list revealed that this list enriched for genes generally expressed in undifferentiated, embryonic tissues but do not necessarily show kidney-specificity (data not shown).

### Identification of nephron-specific genes of different developmental stages

A second protocol was to take advantage of the nephron-deficient *Lim1 *conditional mutant kidneys to identify nephron-specific genes of different developmental stages [20]. To generate an E18.5 nephron-specific gene list, we compared gene expression data of E18.5 control kidney (E18.5C) and *Lim1 *conditional mutant kidney (E18.5M). Fold changes were calculated and used to sort genes. A total of 465 genes showed a more than 2 fold increase in expression in the control kidney compared to the conditional mutant kidney (Table 1 and Additional file 4). The top 50 genes on the list were further evaluated computationally for their kidney specificity. The results indicate that the gene list generated by this protocol is highly enrich for nephron-specific genes. The average relative kidney expression level, based on GNF 2.0, reached 3.5, and the normalized kidney expression level, according to SOURCE, is 32.27%. There is also a slight increase in the ratio of genes with published nephron-specific expression patterns (56%) compared to the gene list generated using the previous protocol (44%). The details of the gene list are described in Table 3.

**Table 3:** Top 50 genes upregulated in the E18.5 control kidney when compared to the *Lim1 *conditional mutant kidney.

Gene Name	Locus Link	Symbol	Fold Change	GNF 2.0	SOURCE (%)	Nephron -specific	Reference
Solute carrier family 34, member 1	20505	Slc34a1	63.47	4.0	78.05		
Uromodulin	22242	Umod	58.19	4.0	61.91	HL,DT	[54]
Calbindin 3	12309	Calb3	45.16	4.0	11.13	DT	[55]
Glutathione S-transferase, alpha 2	14858	Gsta2	41.30	4.0	31.73	PT	[56]
Hydroxyacid oxidase 3	56185	Hao3	40.21	4.0	24.19		
Nephrosis 2 homolog, podocin	170484	Nphs2	39.83	2.0	71.88	Pod	[57]
Kidney-specific membrane protein	57394	Tmem27	37.83	4.0	36.97	DN,DT, PT, UT	[14]
MGC37245	233799	MGC37245	36.09				
Cytochrome P450, family 2, subfamily j, polypeptide 5	13109	Cyp2j5	36.03	4.0	15.60		
FXYD domain-containing ion transport regulator 2	11936	Fxyd2	35.57	4.0	69.12	PT, HL	[58]
Klotho	16591	Kl	35.49	4.0	53.50	PT	[59]
Kidney androgen regulated protein	16483	Kap	35.36	4.0	40.76	PT	[60]
Hydroxysteroid dehydrogenase-4, delta<5>-3-beta	15495	Hsd3b4	34.28	4.0	50.43		
Fructose bisphosphatase 1	14121	Fbp1	32.04	4.0	18.74	DN,DT, PT,UT	[61]
Gamma-glutamyltransferase 1	14598	Ggt1	30.65	4.0	17.68	PT	[62]
Solute carrier family 5, member 8	216225	Slc5a8	29.22		13.49		
Solute carrier family 26, member 1	231583	Slc26a1	27.27	3.0	14.30	PT	[63]
Low density lipoprotein receptor-related protein 2	14725	Lrp2	25.73		51.68	PT,Gl	[64]
Aldolase 2, B isoform	230163	Aldo2	23.80	4.0	37.02	PT	[65]
Kynurenine aminotransferase II	23923	Kat2	23.35	4.0	67.29		
Cubilin	65969	Cubn	22.94	3.0	5.18	PT	[66]
Solute carrier family 12, member 1	20495	Slc12a1	22.45	4.0	74.20	HL	[67]
RIKEN cDNA D630043A20 gene	207151	Slc22a19	21.95	4.0	81.09		
RIKEN cDNA 0610038O04 gene	75396	0610038004Rik	21.91				
Phenylalanine hydroxylase	18478	Pah	21.25	4.0	28.04	PT	[68]
Sulfotransferase family 1D, member 1	53315	Sult1d1	20.47		10.49		
Betaine-homocysteine methyltransferase 2	64918	Bhmt2	20.26	2.5	5.80	PT	[69]
Serine (or cysteine) proteinase inhibitor, clade A, member 6	12401	Serpina6	19.94	0.5	2.56		
Glycine amidinotransferase	67092	Gatm	19.69	4.0	6.83		
Fatty acid binding protein 3	14077	Fabp3	19.60	2.0	4.22		
RIKEN cDNA 9130022A01 gene	100061	9130022A01Rik	18.71	4.0			
UDP-glucuronosyltransferase 1 family, member 2	22236	Ugt1a2	18.42	4.0			
Amnionless	93835	Amn	17.98	3.0	4.01	PT,DT	[14]
Cytochrome P450, family 27, subfamily b, polypeptide 1	13115	Cyp27b1	17.91	0.5	98.60	PT	[70]
Potassium inwardly-rectifying channel, subfamily J, member 15	16516	Kcnj15	17.69		43.57	RT	[70]
Claudin 2	12738	Cldn2	17.10	4.0	8.61	PT	[71]
RIKEN cDNA 2010002A20 gene	66601	2010002A20Rik	16.99	4.0	20.30		
Defensin beta 1	13214	Defb1	16.78	4.0	34.83	CT,HL	[72]
Kidney-derived aspartic protease-like protein	16541	Kdap	16.76	4.0	38.24	PT	[73]
Serine protease inhibitor, Kazal type 3	20730	Spink3	16.30	4.0	1.13		
Hepatic nuclear factor 4	15378	Hnf4	16.20	3.5	5.37	UD,Gl,RV	[74]
Solute carrier family 22, member 6	18399	Slc22a6	15.71	2.5	26.67	DN,PT,Gl	[14]
PDZ domain containing 1	59020	Pdzk1	15.49	4.0	33.70	PT	[75]
Kallikrein 6	16612	Klk6	15.46	4.0	14.16	UT	[76]
RIKEN cDNA 1300013J15 gene	67473	1300013J15Rik	15.36	4.0	32.54		
BB738659		BB738659	15.22				
Alanyl aminopeptidase	16790	Anpep	14.96	2.5	9.70	PT	[77]
Meprin 1 alpha	17287	Mep1a	14.77	3.0	59.06		
Argininosuccinate synthetase 1	11898	Ass1	14.26	4.0	35.65	PT	[78]
Meprin 1 beta	17288	Mep1b	14.13	4.0	2.00		

Abbreviations: CT, collecting tubule; DN, developing nephron; DT: distal convoluted tubule; Gl: glomerulus; HL, loop of Henle; PT, proximal convoluted tubule; Pod, podocyte; RT, renal tubule; RV: renal vesicle; UD, ureteric duct; UT, ureteric tubule.

A gene list enriched for E14.5 nephron-specific genes was generated using the same protocol. Comparison of the E14.5 control kidney and *Lim1 *conditional mutant kidney gene expression profile picked up only 41 genes that showed a more than 2 fold change (Table 1). Unlike the gene list sorted by the comparison made between developmental stages (E14.5C/E18.5C), which does not significantly enrich for E14.5 kidney genes, the comparison between control and *Lim1 *conditional mutant kidney helped to identify kidney-specific genes, especially those expressed in the nephrons. As shown in Table 2, the average relative kidney expression level is as high as 2.9, and there is also a nearly 15 fold increase in the average normalized kidney expression level (21.51 % compare to 1.48%). Thirteen (26%) genes on the top 50 list were also previously described to have a nephron-specific expression pattern. Notably 3 genes related to the Notch signaling pathway, *Msih2*, *Hes5*, and *Jag1 *were found in the list. The details of this gene list are summarized in Table 4.

**Table 4:** Top 41 genes upregulated in the E14.5 control kidney when compared to the *Lim1 *conditional mutant kidney.

Gene Name	Locus Link	Symbol	Fold Change	GNF 2.0	SOURCE (%)	Nephron -specific	Reference
Sulfotransferase family 1D, member 1	53315	Sult1d1	7.22		10.49		
Hydroxyacid oxidase 3	56185	Hao3	7.05	4.0	24.19		
FXYD domain-containing ion transport regulator 2	11936	Fxyd2	5.71	4.0	69.12	PT,HL	[58]
Hepatic nuclear factor 4	15378	Hnf4	5.70	3.5	5.37	UD,DG,RV	[74]
Kidney-specific membrane protein	57394	Tmem27	4.26	4.0	36.97	DN,DT, PT,UT	[14]
Sclerostin domain containing 1	66042	Sostdc1	4.16	3.5	12.36	DT	[79]
Lymphocyte antigen 6 complex, locus A	110454	Ly6a	4.05	2.5	7.10		
AW210596	240638	AW210596	3.38	4.0	5.03		
Aldolase 2, B isoform	230163	Aldo2	3.27	4.0	37.02	PT	[65]
RIKEN cDNA 9930038N01 gene		9930038N01Rik	3.13				
RIKEN cDNA 0610033E06 gene	112417	0610033E06Rik	3.07	3.5	37.77		
Musashi homolog 2	76626	Msi2h	2.87		1.12		
ATPase, Na+/K+ transporting, beta 1 polypeptide	11931	Atp1b1	2.83	2.5	8.64		
Hairy and enhancer of split 5	15208	Hes5	2.67	0.5	0.00	CB,SB, PT	[40]
RIKEN cDNA 1300013J15 gene	67473	1300013J15Rik	2.55	4.0	32.54		
BC013481	245945	BC013481	2.51		1.72		
PDZ domain containing 1	59020	Pdzk1	2.51	4.0	33.70	PT	[75]
RIKEN cDNA D630042F21 gene	330428	D630042F21Rik	2.50		70.39		
Glucosaminyl (N-acetyl) transferase 1, core 2	14537	Gcnt1	2.45		5.38		
Dipeptidylpeptidase 4	13482	Dpp4	2.38			PT,DT	[80]
Low density lipoprotein receptor-related protein 2	14725	Lrp2	2.37		51.68	PT,Gl	[64]
Purkinje cell protein 4	14858	Pcp4	2.35	-2.0	0.00		
Glutathione S-transferase, alpha 2	18546	Gsta2	2.35	4.0	31.73	PT	[56]
MGC37245	233799	MGC37245	2.29				
BB427389		BB427389	2.28				
Single-minded 1	20464	Sim1	2.27	2.5	0.00	RT	81
Kidney expressed gene 1	64697	Keg1	2.26	4.0	49.76		
BG064527		BG064527	2.24				
RIKEN cDNA 2610511G16 gene	67500	2610511G16Rik	2.20				
Nephrosis 2 homolog, podocin	170484	Nphs2	2.20	2.0	71.88	Pod	57
Solute carrier family 34, member 1	20505	Slc34a1	2.16	4.0	78.05		
RIKEN cDNA 2700008B19 gene	217026	2700008B19Rik	2.15		1.76		
RIKEN cDNA 6332401O19 gene	319832	6332401O19Rik	2.14		6.71		
RIKEN cDNA 9130423L19 gene	74570	9130423L19Rik	2.11	1.5			
C85657		C85657	2.11				
RIKEN cDNA 5730493B19 gene		5730493B19Rik	2.10				
Fucosyltransferase 9	14348	Fut9	2.10	4.0	3.15		
AU022045		AU022045	2.07				
Jagged 1	16449	Jag1	2.06	1.5	1.20	RV, CB, SB,PT	[82]
EH-domain containing 2	259300	Ehd2	2.02		0.00		
RIKEN cDNA 2610510D13 gene	229279	2610510D13Rik	2.01	-0.5			

Abbreviations: CB, comma-shaped body; CT, collecting tubule; DN, developing nephron; DG, developing glomerulus; DT: distal convoluted tubule; Gl: glomerulus; HL, loop of Henle; PT, proximal convoluted tubule; Pod, podocyte; RT, renal tubule; RV: renal vesicle; SB, S-shaped body; UD, ureteric duct; UT, ureteric tubule

### Ontological analysis on nephron-specific genes of different developmental stages

**Figure 1:**
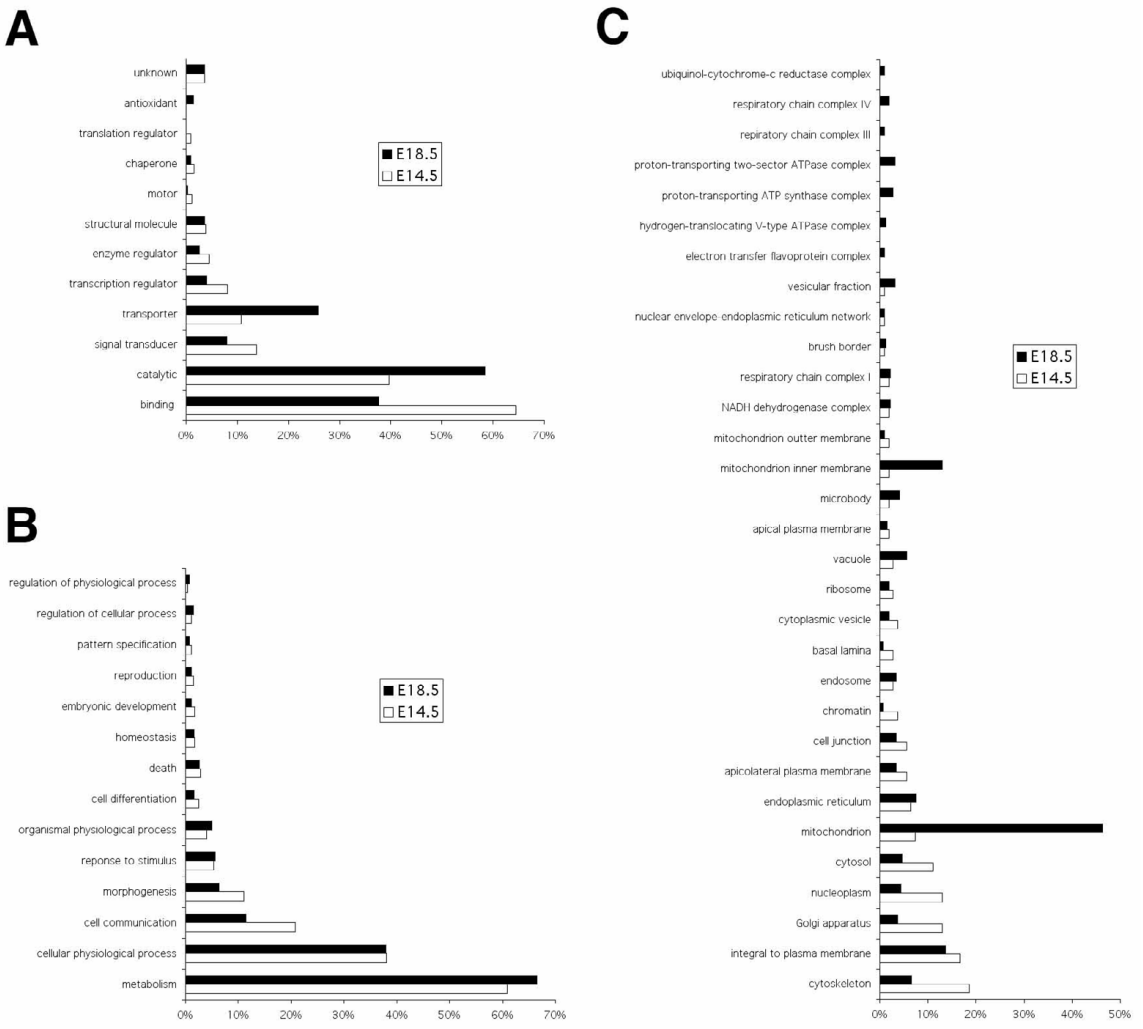
**Ontological analyses of the top 1,000 genes upregulated in E18.5 and E14.5 control kidneys**. A. Molecular Function Level 1 analysis (annotation rates: E18.5 – 63.6%, E14.5 – 33.5%). B. Biological Process Level 2 analysis (annotation rates: E18.5 – 54.0%, E14.5 – 27.9%). C. Cell Component Level 4 analysis (annotation rates of the top 1,000 genes: E18.5 – 32.2%, E14.5 – 10.8%). Only molecule categories containing at least 3 hits in either of the gene lists are shown. The numbers of genes fell in major functional categories were normalized by the numbers of gene annotated in each list and were expressed in percentages.

To gain insight into the functional aspects of the microarray data, we exploited the web-based annotation tool, DAVID, to help identify functional themes that showed differences between the control kidney and the *Lim1 *conditional mutant kidney [36]. Each of the top 1,000 genes on the lists that displayed upregulation in the control kidney compared to the *Lim1 *conditional mutant kidney at either E18.5 or E14.5 were used for this analysis. Ontological analyses were performed at Molecular Function Level 1, Biological Process Level 2, and Cell Component Level 4 [35]. The results of major functional categories are shown in Figure 1. The numbers of genes that fell in major categories were normalized by the number of genes annotated in each list and were expressed in percentages. Generally speaking nephron-specific genes identified at E18.5 were better studied. Close to 60% of these genes were annotated in Molecular Function and Biological Process ontologies and 32% in Cell Component at the levels our analyses were performed. In contrast, only about 30% and 11% of the genes on the E14.5 nephron-specific gene list were annotated at the same levels.

Comparisons made at Molecular Function Level 1 (Figure 1A) revealed that the majority of the E18.5 nephron genes identified in our screen were described to possess catalytic activity (58.49%) whereas molecules found in the E14.5 nephron gene list were better studied for their physical interactions with other molecules (e.g. binding, 64.48%). The E18.5 gene list is also characterized by a relatively high proportion of transporter proteins (25.79%) whereas the E14.5 gene list contains a higher ratio of genes with signal transducer (13.73%), transcription regulator (8.06%), enzyme regulator (4.48%), motor (1.19%) and translation regulator (0.90%) functions.

Ontological analysis at Biological Process Level 2 (Figure 1B) indicated that the E14.5 nephron-specific gene list favors molecules involved in cell communication (20.79%) and morphogenesis (11.11%). Metabolism is detected as a very significant functional theme in the E18.5 gene list with an EASE score of 7.72 E-5 (data not shown) [37].

Cell Component Level 4 ontological study (Figure 1C) of E18.5 nephron-specific genes featured by a high proportion of genes identified at subcellular sites related to energy metabolism, including mitochondria (46.27%), mitochodrial inner membrane (13.04%), electron transfer flavoprotein complex (0.93%), hydrogen-translocating V-type ATPase complex (1.24%), proton-transporting ATP synthase complex (2.80%), proton-transporting two-sector ATPase complex (3.11%), respiratory chain complex III (0.93%), respiratory chain complex IV (1.86%), and ubiquinol-cytochrome-c reductase complex (0.93%). There were also more proteins found at the vacuole (5.59%), microbody (4.04%) and vesicular fraction (3.11%). However, a higher proportion of proteins in the cytoskeleton (18.52%), Golgi apparatus (12.96%), nucleoplasm (12.96%), cytosol (11.11%), chromatin (3.70%) and basal lamina (2.78%) was found in the E14.5 gene list.

## Discussion

The mouse is one of the most widely used animal models to study human biology especially after the development of embryonic stem (ES) cells and the assembly and annotation of the mouse genome sequence [2-4,38]. ES cells and gene targeting technology allow the construction of transgenic mice with defined genetic modifications. The availability of whole genome sequences forms the basis of the development of high-throughput technologies, such as microarrays, to conduct research at a genomic level. Since it is relatively difficult to collect significant numbers of genetically well-defined human samples, it is important to perform research on an evolutionarily close species prior to their human applications. In this study, we took advantage of the nephron-deficient kidneys from metanephric mesenchyme-specific *Lim1 *conditional mutant mice to perform a genome-wide screen for developing nephron genes. Whereas similar studies have been performed on kidneys from meprin β, vitamin D receptor, aquaporin-1, or metallothionein knockout mice, our study is the first to use a tissue-specific approach in the kidney [15-18].

Computational analysis and PubMed search suggested that the expression profile comparison between control and *Lim1 *conditional mutant kidneys generated gene lists enriched for nephron-specific genes. In global gene expression level studies, ribosomal genes and other housekeeping genes that are highly expressed but do not show any tissue- or developmental stage-specificity are always identified. In this study, we used two different protocols to enrich for developmental stage-specific kidney genes and nephron-specific genes of different developmental stages. Two independent online gene expression databases, namely GNF Expression Atlas 2.0 and Unigene were used to evaluate the tissue-specific enrichment computationally. Our results indicated dramatic enrichments for kidney-specific genes by both protocols in the E18.5 experiments. However, the comparison made between E14.5 and E18.5 control kidneys did not generate a kidney-specific gene list. In our opinion, there are at least two possible reasons. Firstly, data from the two online databases we used were based on experiments performed using either adult or neonate kidney tissues. Neither of them are likely to reflect gene expression profiles during early nephron development. Secondly, since many important molecular pathways and fundamental developmental processes are repeatedly observed in different organ systems, genes predominantly expressed during early nephron development are also likely to be found in other undifferentiated tissues. A closer look of the gene list results in a conclusion consistent with this latter assumption. Many of the genes identified by this comparison are commonly found in undifferentiated tissues (data not shown). In contrast, the comparison between E14.5 control and *Lim1 *conditional mutant kidneys identified molecules that are also found in mature nephrons, which predominantly contributed to the kidney-specificity in our evaluation, and molecules involved in early nephron development. For example, *podocin *(*Nphs2*) is only expressed in terminally differentiated podocytes [39]. Elevated podocin level observed in E14.5 control kidney suggests that the first podocyte is found before or around E14.5. Notably, regulatory genes important in early nephron development, such as *Msi2h*, *Hes5*, and *Jag1*, which are involved in Notch signaling [40-45], do not show kidney-specificity based on GNF Atlas 2.0 and UniGene data. However, our screening protocol placed them on top of our list. Therefore, the use of *Lim1 *conditional mutant tissue as an RNA source in our microarray experiment helped to identify nephron developmental genes.

The results of our ontological comparison made between the E18.5 and E14.5 nephron-specific gene lists are consistent with current concepts of kidney organogenesis and a previous study [46,47]. Gene ontology (GO) annotations provide structured, precisely defined, common, controlled vocabulary for describing the roles of genes and their products in any organism. It is the current representation of biological knowledge as well as serving as a guide for organizing new data [35]. However, one should keep in mind that the gene ontology is a dynamic, web-based resource, the annotations are not complete and their accuracy is limited by current knowledge of the molecules. Although kidney organogenesis is a continuous process, the first nephron is not seen until around E16.5 [1,23]. Nephrons forming in a E14.5 kidney are mainly composed of reciprocally induced tissues, stem cell growth and differentiation, cell polarization, mesenchyme to epithelia transformation, branching morphogenesis, angiogenesis, apoptosis, proximal-distal segmentation and the differentiation of several interesting cell types. In our oncological analysis, E14.5 forming nephron-specific genes are composed of those better studied for their protein-protein interaction (binding) and possess signal transducer, transcription regulator and enzyme regulator activities. A higher portion of them are involved in cell communication and morphogenesis process. Interestingly, they are associated with cytoskeleton and nuclear compartments (nucleoplasm and chromatin). In contrast, there are many mature nephrons present in an E18.5 kidney. Therefore, we were expecting to observe genes related to kidney function, e.g. those involved in solute transport and energy metabolism. Our results indicate a relatively high number of E18.5 nephron-specific genes possess catalytic activity (presumably related to energy metabolism and the extensive extracellular matrix change in late kidney development) and exert their function as transporters. Consistently, an extremely high proportion of them encoded proteins located in the mitochondria.

Ontological analysis and a detailed examination of the top 1,000 E14.5 nephron-specific gene list suggest that genes with modest upregulation in the control kidney (fold changes less than 2 in our experiment) are also interesting. For example, *Brn1 *(1.34) and *EphA4 *(1.67) were previously shown to be downstream of *Lim1 *[20,25]. *Fzd4 *(1.71) has been considered a candidate receptor to transduce Wnt4 signals during kidney organogenesis [1,48]. *Irx2 *(1.52) and *Irx3 *(1.28) are homeobox genes previously reported to be expressed in the developing nephrons [49]. *Crb3 *(1.40) is known to localize to kidney epithelia and is essential for ciliogenesis [50,51]. The top 1,000 genes upregulated in the E14.5 control kidney is supplied in Additional file 5.

Tissue heterogeniety always complicates the interpretation of microarray data although analyses of different organs or even on organisms of different developmental stages have been reported [30,52]. In our experiment, we used the whole kidney as a tissue source for RNA preparation. The complexity of kidney structure and development limits interpretations of our results. Improvements in tissue collection methods such as laser capture microdissection and fluoresence-activated cell sorting (FACS) on genetic marked/fluorescent protein-labelled transgenic tissue have been developed [14,53] and could provide more specificity. Nevertheless, our study demonstrates that genetically engineered mouse organs can be used to identify tissue-specific and developmentally regulated genes during mammalian organogenesis.

## Conclusion

Our experimental results indicate that the expression profile comparisons between the control and the *Lim1 *conditional mutant kidneys generated nephron-specific gene lists. Our results demonstrate the feasibility of exploiting genetically engineered kidneys to identify developing nephron-specific genes.

## Competing interests

The author(s) declare that they have no competing interests.

## Authors' contributions

YTC designed the study, carried out sample collection, performed computational analysis, and prepared the manuscript. AK conceived the study, participated in sample collection, and participated in discussions. KMK helped in sample collection and contributed to the discussion. RLJ provided microarray chips and contributed to the discussion. RRB participated in study design, supervised data collection, and prepared the manuscript.

## Pre-publication history

The pre-publication history for this paper can be accessed here:



## Supplementary Material

Additional file 1 Expression data for all the microarrays used in this study. Click here for file

Additional file 2 **E18.5 kidney gene list**. Sorted by E18.5C/E14.5C; 1,006 genes showed a more than 2 fold change. Click here for file

Additional file 3 **E14.5 kidney gene list**. Sorted by E14.5C/E18.5C; 796 genes showed a more than 2 fold change. Click here for file

Additional file 4 **E18.5 nephron gene list**. Sorted by E18.5C/E18.5M; 465 genes showed a more than 2fold change; top 1,000 genes. Click here for file

Additional file 5 **E14.5 nephron gene list**. Sorted by E14.5C/E14.5M; 41 genes showed a more than 2 fold change; top 1,000 genes. Click here for file

## References

[B1] Vainio S, Lin Y (2002). Coordinating early kidney development: lessons from gene targeting. Nat Rev Genet.

[B2] Waterston RH, Lindblad-Toh K, Birney E, Rogers J, Abril JF, Agarwal P, Agarwala R, Ainscough R, Alexandersson M, An P, Antonarakis SE, Attwood J, Baertsch R, Bailey J, Barlow K, Beck S, Berry E, Birren B, Bloom T, Bork P, Botcherby M, Bray N, Brent MR, Brown DG, Brown SD, Bult C, Burton J, Butler J, Campbell RD, Carninci P, Cawley S, Chiaromonte F, Chinwalla AT, Church DM, Clamp M, Clee C, Collins FS, Cook LL, Copley RR, Coulson A, Couronne O, Cuff J, Curwen V, Cutts T, Daly M, David R, Davies J, Delehaunty KD, Deri J, Dermitzakis ET, Dewey C, Dickens NJ, Diekhans M, Dodge S, Dubchak I, Dunn DM, Eddy SR, Elnitski L, Emes RD, Eswara P, Eyras E, Felsenfeld A, Fewell GA, Flicek P, Foley K, Frankel WN, Fulton LA, Fulton RS, Furey TS, Gage D, Gibbs RA, Glusman G, Gnerre S, Goldman N, Goodstadt L, Grafham D, Graves TA, Green ED, Gregory S, Guigo R, Guyer M, Hardison RC, Haussler D, Hayashizaki Y, Hillier LW, Hinrichs A, Hlavina W, Holzer T, Hsu F, Hua A, Hubbard T, Hunt A, Jackson I, Jaffe DB, Johnson LS, Jones M, Jones TA, Joy A, Kamal M, Karlsson EK, Karolchik D, Kasprzyk A, Kawai J, Keibler E, Kells C, Kent WJ, Kirby A, Kolbe DL, Korf I, Kucherlapati RS, Kulbokas EJ, Kulp D, Landers T, Leger JP, Leonard S, Letunic I, Levine R, Li J, Li M, Lloyd C, Lucas S, Ma B, Maglott DR, Mardis ER, Matthews L, Mauceli E, Mayer JH, McCarthy M, McCombie WR, McLaren S, McLay K, McPherson JD, Meldrim J, Meredith B, Mesirov JP, Miller W, Miner TL, Mongin E, Montgomery KT, Morgan M, Mott R, Mullikin JC, Muzny DM, Nash WE, Nelson JO, Nhan MN, Nicol R, Ning Z, Nusbaum C, O'Connor MJ, Okazaki Y, Oliver K, Overton-Larty E, Pachter L, Parra G, Pepin KH, Peterson J, Pevzner P, Plumb R, Pohl CS, Poliakov A, Ponce TC, Ponting CP, Potter S, Quail M, Reymond A, Roe BA, Roskin KM, Rubin EM, Rust AG, Santos R, Sapojnikov V, Schultz B, Schultz J, Schwartz MS, Schwartz S, Scott C, Seaman S, Searle S, Sharpe T, Sheridan A, Shownkeen R, Sims S, Singer JB, Slater G, Smit A, Smith DR, Spencer B, Stabenau A, Stange-Thomann N, Sugnet C, Suyama M, Tesler G, Thompson J, Torrents D, Trevaskis E, Tromp J, Ucla C, Ureta-Vidal A, Vinson JP, Von Niederhausern AC, Wade CM, Wall M, Weber RJ, Weiss RB, Wendl MC, West AP, Wetterstrand K, Wheeler R, Whelan S, Wierzbowski J, Willey D, Williams S, Wilson RK, Winter E, Worley KC, Wyman D, Yang S, Yang SP, Zdobnov EM, Zody MC, Lander ES (2002). Initial sequencing and comparative analysis of the mouse genome. Nature.

[B3] Okazaki Y, Furuno M, Kasukawa T, Adachi J, Bono H, Kondo S, Nikaido I, Osato N, Saito R, Suzuki H, Yamanaka I, Kiyosawa H, Yagi K, Tomaru Y, Hasegawa Y, Nogami A, Schonbach C, Gojobori T, Baldarelli R, Hill DP, Bult C, Hume DA, Quackenbush J, Schriml LM, Kanapin A, Matsuda H, Batalov S, Beisel KW, Blake JA, Bradt D, Brusic V, Chothia C, Corbani LE, Cousins S, Dalla E, Dragani TA, Fletcher CF, Forrest A, Frazer KS, Gaasterland T, Gariboldi M, Gissi C, Godzik A, Gough J, Grimmond S, Gustincich S, Hirokawa N, Jackson IJ, Jarvis ED, Kanai A, Kawaji H, Kawasawa Y, Kedzierski RM, King BL, Konagaya A, Kurochkin IV, Lee Y, Lenhard B, Lyons PA, Maglott DR, Maltais L, Marchionni L, McKenzie L, Miki H, Nagashima T, Numata K, Okido T, Pavan WJ, Pertea G, Pesole G, Petrovsky N, Pillai R, Pontius JU, Qi D, Ramachandran S, Ravasi T, Reed JC, Reed DJ, Reid J, Ring BZ, Ringwald M, Sandelin A, Schneider C, Semple CA, Setou M, Shimada K, Sultana R, Takenaka Y, Taylor MS, Teasdale RD, Tomita M, Verardo R, Wagner L, Wahlestedt C, Wang Y, Watanabe Y, Wells C, Wilming LG, Wynshaw-Boris A, Yanagisawa M, Yang I, Yang L, Yuan Z, Zavolan M, Zhu Y, Zimmer A, Carninci P, Hayatsu N, Hirozane-Kishikawa T, Konno H, Nakamura M, Sakazume N, Sato K, Shiraki T, Waki K, Kawai J, Aizawa K, Arakawa T, Fukuda S, Hara A, Hashizume W, Imotani K, Ishii Y, Itoh M, Kagawa I, Miyazaki A, Sakai K, Sasaki D, Shibata K, Shinagawa A, Yasunishi A, Yoshino M, Waterston R, Lander ES, Rogers J, Birney E, Hayashizaki Y (2002). Analysis of the mouse transcriptome based on functional annotation of 60,770 full-length cDNAs. Nature.

[B4] Kawai J, Shinagawa A, Shibata K, Yoshino M, Itoh M, Ishii Y, Arakawa T, Hara A, Fukunishi Y, Konno H, Adachi J, Fukuda S, Aizawa K, Izawa M, Nishi K, Kiyosawa H, Kondo S, Yamanaka I, Saito T, Okazaki Y, Gojobori T, Bono H, Kasukawa T, Saito R, Kadota K, Matsuda H, Ashburner M, Batalov S, Casavant T, Fleischmann W, Gaasterland T, Gissi C, King B, Kochiwa H, Kuehl P, Lewis S, Matsuo Y, Nikaido I, Pesole G, Quackenbush J, Schriml LM, Staubli F, Suzuki R, Tomita M, Wagner L, Washio T, Sakai K, Okido T, Furuno M, Aono H, Baldarelli R, Barsh G, Blake J, Boffelli D, Bojunga N, Carninci P, de Bonaldo MF, Brownstein MJ, Bult C, Fletcher C, Fujita M, Gariboldi M, Gustincich S, Hill D, Hofmann M, Hume DA, Kamiya M, Lee NH, Lyons P, Marchionni L, Mashima J, Mazzarelli J, Mombaerts P, Nordone P, Ring B, Ringwald M, Rodriguez I, Sakamoto N, Sasaki H, Sato K, Schonbach C, Seya T, Shibata Y, Storch KF, Suzuki H, Toyo-oka K, Wang KH, Weitz C, Whittaker C, Wilming L, Wynshaw-Boris A, Yoshida K, Hasegawa Y, Kawaji H, Kohtsuki S, Hayashizaki Y (2001). Functional annotation of a full-length mouse cDNA collection. Nature.

[B5] Schwab K, Patterson LT, Aronow BJ, Luckas R, Liang HC, Potter SS (2003). A catalogue of gene expression in the developing kidney. Kidney Int.

[B6] Yoshida T, Muller E, Stears R, Shirota S, Tsuchiya K, Akiba T, Gullans SR (2004). Osmoadaptation-related genes in inner medulla of mouse kidney using microarray. Biochem Biophys Res Commun.

[B7] Stuart RO, Bush KT, Nigam SK (2003). Changes in gene expression patterns in the ureteric bud and metanephric mesenchyme in models of kidney development. Kidney Int.

[B8] Valerius MT, Patterson LT, Witte DP, Potter SS (2002). Microarray analysis of novel cell lines representing two stages of metanephric mesenchyme differentiation. Mech Dev.

[B9] Supavekin S, Zhang W, Kucherlapati R, Kaskel FJ, Moore LC, Devarajan P (2003). Differential gene expression following early renal ischemia/reperfusion. Kidney Int.

[B10] Gumz ML, Popp MP, Wingo CS, Cain BD (2003). Early transcriptional effects of aldosterone in a mouse inner medullary collecting duct cell line. Am J Physiol Renal Physiol.

[B11] Kieran NE, Doran PP, Connolly SB, Greenan MC, Higgins DF, Leonard M, Godson C, Taylor CT, Henger A, Kretzler M, Burne MJ, Rabb H, Brady HR (2003). Modification of the transcriptomic response to renal ischemia/reperfusion injury by lipoxin analog. Kidney Int.

[B12] Susztak K, Bottinger E, Novetsky A, Liang D, Zhu Y, Ciccone E, Wu D, Dunn S, McCue P, Sharma K (2004). Molecular profiling of diabetic mouse kidney reveals novel genes linked to glomerular disease. Diabetes.

[B13] Kim JH, Ha IS, Hwang CI, Lee YJ, Kim J, Yang SH, Kim YS, Cao YA, Choi S, Park WY (2004). Gene expression profiling of anti-GBM glomerulonephritis model: the role of NF-kappaB in immune complex kidney disease. Kidney Int.

[B14] Takasato M, Osafune K, Matsumoto Y, Kataoka Y, Yoshida N, Meguro H, Aburatani H, Asashima M, Nishinakamura R (2004). Identification of kidney mesenchymal genes by a combination of microarray analysis and Sall1-GFP knockin mice. Mech Dev.

[B15] Norman LP, Jiang W, Han X, Saunders TL, Bond JS (2003). Targeted disruption of the meprin beta gene in mice leads to underrepresentation of knockout mice and changes in renal gene expression profiles. Mol Cell Biol.

[B16] Li X, Zheng W, Li YC (2003). Altered gene expression profile in the kidney of vitamin D receptor knockout mice. J Cell Biochem.

[B17] McReynolds MR, Taylor-Garcia KM, Greer KA, Hoying JB, Brooks HL (2005). Renal medullary gene expression in aquaporin-1 null mice. Am J Physiol Renal Physiol.

[B18] Miura N, Koizumi S (2005). Gene expression profiles in the liver and kidney of metallothionein-null mice. Biochem Biophys Res Commun.

[B19] Karavanov AA, Karavanova I, Perantoni A, Dawid IB (1998). Expression pattern of the rat Lim-1 homeobox gene suggests a dual role during kidney development. Int J Dev Biol.

[B20] Kobayashi A, Kwan KM, Carroll TJ, McMahon AP, Mendelsohn CL, Behringer RR (2005). Distinct and sequential tissue-specific activities of the LIM-class homeobox gene Lim1 for tubular morphogenesis during kidney development. Development.

[B21] Shawlot W, Behringer RR (1995). Requirement for Lim1 in head-organizer function. Nature.

[B22] Kwan KM, Behringer RR (2002). Conditional inactivation of Lim1 function. Genesis.

[B23] Kaufman MH, Bard JBL (1999). The anatomical basis of mouse development.

[B24] Wang P, Pereira FA, Beasley D, Zheng H (2003). Presenilins are required for the formation of comma- and S-shaped bodies during nephrogenesis. Development.

[B25] Kania A, Johnson RL, Jessell TM (2000). Coordinate roles for LIM homeobox genes in directing the dorsoventral trajectory of motor axons in the vertebrate limb. Cell.

[B26] Li C, Wong WH (2001). Model-based analysis of oligonucleotide arrays: expression index computation and outlier detection. Proc Natl Acad Sci U S A.

[B27] Schadt EE, Li C, Ellis B, Wong WH (2001). Feature extraction and normalization algorithms for high-density oligonucleotide gene expression array data. J Cell Biochem Suppl.

[B28] Brazma A, Hingamp P, Quackenbush J, Sherlock G, Spellman P, Stoeckert C, Aach J, Ansorge W, Ball CA, Causton HC, Gaasterland T, Glenisson P, Holstege FC, Kim IF, Markowitz V, Matese JC, Parkinson H, Robinson A, Sarkans U, Schulze-Kremer S, Stewart J, Taylor R, Vilo J, Vingron M (2001). Minimum information about a microarray experiment (MIAME)-toward standards for microarray data. Nat Genet.

[B29] UCSC Mouse Gene Sorter. http://genome.ucsc.edu/cgi-bin/hgNear.

[B30] Su AI, Wiltshire T, Batalov S, Lapp H, Ching KA, Block D, Zhang J, Soden R, Hayakawa M, Kreiman G, Cooke MP, Walker JR, Hogenesch JB (2004). A gene atlas of the mouse and human protein-encoding transcriptomes. Proc Natl Acad Sci U S A.

[B31] Wheeler DL, Barrett T, Benson DA, Bryant SH, Canese K, Church DM, DiCuccio M, Edgar R, Federhen S, Helmberg W, Kenton DL, Khovayko O, Lipman DJ, Madden TL, Maglott DR, Ostell J, Pontius JU, Pruitt KD, Schuler GD, Schriml LM, Sequeira E, Sherry ST, Sirotkin K, Starchenko G, Suzek TO, Tatusov R, Tatusova TA, Wagner L, Yaschenko E (2005). Database resources of the National Center for Biotechnology Information. Nucleic Acids Res.

[B32] SOURCE. http://source.stanford.edu.

[B33] Diehn M, Sherlock G, Binkley G, Jin H, Matese JC, Hernandez-Boussard T, Rees CA, Cherry JM, Botstein D, Brown PO, Alizadeh AA (2003). SOURCE: a unified genomic resource of functional annotations, ontologies, and gene expression data. Nucleic Acids Res.

[B34] Database for Annotation, Visualization and Integrated Discovery. http://apps1.niaid.nih.gov/DAVID.

[B35] Ashburner M, Ball CA, Blake JA, Botstein D, Butler H, Cherry JM, Davis AP, Dolinski K, Dwight SS, Eppig JT, Harris MA, Hill DP, Issel-Tarver L, Kasarskis A, Lewis S, Matese JC, Richardson JE, Ringwald M, Rubin GM, Sherlock G (2000). Gene ontology: tool for the unification of biology. The Gene Ontology Consortium. Nat Genet.

[B36] Dennis G, Sherman BT, Hosack DA, Yang J, Gao W, Lane HC, Lempicki RA (2003). DAVID: Database for Annotation, Visualization, and Integrated Discovery. Genome Biol.

[B37] Hosack DA, Dennis G, Sherman BT, Lane HC, Lempicki RA (2003). Identifying biological themes within lists of genes with EASE. Genome Biol.

[B38] Bradley A, Evans M, Kaufman MH, Robertson E (1984). Formation of germ-line chimaeras from embryo-derived teratocarcinoma cell lines. Nature.

[B39] Miner JH (2002). Focusing on the glomerular slit diaphragm: podocin enters the picture. Am J Pathol.

[B40] Piscione TD, Wu MY, Quaggin SE (2004). Expression of Hairy/Enhancer of Split genes, Hes1 and Hes5, during murine nephron morphogenesis. Gene Expr Patterns.

[B41] McCright B (2003). Notch signaling in kidney development. Curr Opin Nephrol Hypertens.

[B42] McCright B, Lozier J, Gridley T (2002). A mouse model of Alagille syndrome: Notch2 as a genetic modifier of Jag1 haploinsufficiency. Development.

[B43] McCright B, Gao X, Shen L, Lozier J, Lan Y, Maguire M, Herzlinger D, Weinmaster G, Jiang R, Gridley T (2001). Defects in development of the kidney, heart and eye vasculature in mice homozygous for a hypomorphic Notch2 mutation. Development.

[B44] Li L, Krantz ID, Deng Y, Genin A, Banta AB, Collins CC, Qi M, Trask BJ, Kuo WL, Cochran J, Costa T, Pierpont ME, Rand EB, Piccoli DA, Hood L, Spinner NB (1997). Alagille syndrome is caused by mutations in human Jagged1, which encodes a ligand for Notch1. Nat Genet.

[B45] Okano H, Imai T, Okabe M (2002). Musashi: a translational regulator of cell fate. J Cell Sci.

[B46] Saxen L (1987). Organogenesis of the Kidney.

[B47] Stuart RO, Bush KT, Nigam SK (2001). Changes in global gene expression patterns during development and maturation of the rat kidney. Proc Natl Acad Sci U S A.

[B48] Stark MR, Rao MS, Schoenwolf GC, Yang G, Smith D, Mauch TJ (2000). Frizzled-4 expression during chick kidney development. Mech Dev.

[B49] Houweling AC, Dildrop R, Peters T, Mummenhoff J, Moorman AF, Ruther U, Christoffels VM (2001). Gene and cluster-specific expression of the Iroquois family members during mouse development. Mech Dev.

[B50] Lemmers C, Michel D, Lane-Guermonprez L, Delgrossi MH, Medina E, Arsanto JP, Le Bivic A (2004). CRB3 binds directly to Par6 and regulates the morphogenesis of the tight junctions in mammalian epithelial cells. Mol Biol Cell.

[B51] Makarova O, Roh MH, Liu CJ, Laurinec S, Margolis B (2003). Mammalian Crumbs3 is a small transmembrane protein linked to protein associated with Lin-7 (Pals1). Gene.

[B52] White KP, Rifkin SA, Hurban P, Hogness DS (1999). Microarray analysis of Drosophila development during metamorphosis. Science.

[B53] Mikulowska-Mennis A, Taylor TB, Vishnu P, Michie SA, Raja R, Horner N, Kunitake ST (2002). High-quality RNA from cells isolated by laser capture microdissection. Biotechniques.

[B54] Zhu X, Cheng J, Gao J, Lepor H, Zhang ZT, Pak J, Wu XR (2002). Isolation of mouse THP gene promoter and demonstration of its kidney-specific activity in transgenic mice. Am J Physiol Renal Physiol.

[B55] Li H, Christakos S (1991). Differential regulation by 1,25-dihydroxyvitamin D3 of calbindin-D9k and calbindin-D28k gene expression in mouse kidney. Endocrinology.

[B56] Rabahi F, Brule S, Sirois J, Beckers JF, Silversides DW, Lussier JG (1999). High expression of bovine alpha glutathione S-transferase (GSTA1, GSTA2) subunits is mainly associated with steroidogenically active cells and regulated by gonadotropins in bovine ovarian follicles. Endocrinology.

[B57] Moeller MJ, Sanden SK, Soofi A, Wiggins RC, Holzman LB (2002). Two gene fragments that direct podocyte-specific expression in transgenic mice. J Am Soc Nephrol.

[B58] Jones DH, Golding MC, Barr KJ, Fong GH, Kidder GM (2001). The mouse Na+-K+-ATPase gamma-subunit gene (Fxyd2) encodes three developmentally regulated transcripts. Physiol Genomics.

[B59] Kato Y, Arakawa E, Kinoshita S, Shirai A, Furuya A, Yamano K, Nakamura K, Iida A, Anazawa H, Koh N, Iwano A, Imura A, Fujimori T, Kuro-o M, Hanai N, Takeshige K, Nabeshima Y (2000). Establishment of the anti-Klotho monoclonal antibodies and detection of Klotho protein in kidneys. Biochem Biophys Res Commun.

[B60] Meseguer A, Catterall JF (1987). Mouse kidney androgen-regulated protein messenger ribonucleic acid is expressed in the proximal convoluted tubules. Mol Endocrinol.

[B61] Stein S, Liehr T, Eschrich K (2001). Characterization of the mouse liver fructose-1,6-bisphosphatase gene. Gene.

[B62] Darbouy M, Chobert MN, Lahuna O, Okamoto T, Bonvalet JP, Farman N, Laperche Y (1991). Tissue-specific expression of multiple gamma-glutamyl transpeptidase mRNAs in rat epithelia. Am J Physiol.

[B63] Lee A, Beck L, Markovich D (2003). The mouse sulfate anion transporter gene Sat1 (Slc26a1): cloning, tissue distribution, gene structure, functional characterization, and transcriptional regulation thyroid hormone. DNA Cell Biol.

[B64] Oleinikov AV, Makker SP (2000). Increased expression of cytoplasmic tail-containing form of gp600/megalin in active Heymann nephritis. J Pathol.

[B65] Vallet V, Bens M, Antoine B, Levrat F, Miquerol L, Kahn A, Vandewalle A (1995). Transcription factors and aldolase B gene expression in microdissected renal proximal tubules and derived cell lines. Exp Cell Res.

[B66] Christensen EI, Verroust PJ (2002). Megalin and cubilin, role in proximal tubule function and during development. Pediatr Nephrol.

[B67] Igarashi P, Vanden Heuvel GB, Payne JA, Forbush B (1995). Cloning, embryonic expression, and alternative splicing of a murine kidney-specific Na-K-Cl cotransporter. Am J Physiol.

[B68] Lichter-Konecki U, Hipke CM, Konecki DS (1999). Human phenylalanine hydroxylase gene expression in kidney and other nonhepatic tissues. Mol Genet Metab.

[B69] Delgado-Reyes CV, Wallig MA, Garrow TA (2001). Immunohistochemical detection of betaine-homocysteine S-methyltransferase in human, pig, and rat liver and kidney. Arch Biochem Biophys.

[B70] Zhang MY, Wang X, Wang JT, Compagnone NA, Mellon SH, Olson JL, Tenenhouse HS, Miller WL, Portale AA (2002). Dietary phosphorus transcriptionally regulates 25-hydroxyvitamin D-1alpha-hydroxylase gene expression in the proximal renal tubule. Endocrinology.

[B71] Enck AH, Berger UV, Yu AS (2001). Claudin-2 is selectively expressed in proximal nephron in mouse kidney. Am J Physiol Renal Physiol.

[B72] Schnapp D, Reid CJ, Harris A (1998). Localization of expression of human beta defensin-1 in the pancreas and kidney. J Pathol.

[B73] Mori K, Ogawa Y, Tamura N, Ebihara K, Aoki T, Muro S, Ozaki S, Tanaka I, Tashiro K, Nakao K (1997). Molecular cloning of a novel mouse aspartic protease-like protein that is expressed abundantly in the kidney. FEBS Lett.

[B74] Taraviras S, Monaghan AP, Schutz G, Kelsey G (1994). Characterization of the mouse HNF-4 gene and its expression during mouse embryogenesis. Mech Dev.

[B75] Capuano P, Bacic D, Stange G, Hernando N, Kaissling B, Pal R, Kocher O, Biber J, Wagner CA, Murer H (2005). Expression and regulation of the renal Na/phosphate cotransporter NaPi-IIa in a mouse model deficient for the PDZ protein PDZK1. Pflugers Arch.

[B76] Petraki CD, Karavana VN, Skoufogiannis PT, Little SP, Howarth DJ, Yousef GM, Diamandis EP (2001). The spectrum of human kallikrein 6 (zyme/protease M/neurosin) expression in human tissues as assessed by immunohistochemistry. J Histochem Cytochem.

[B77] Yang XF, Milhiet PE, Gaudoux F, Crine P, Boileau G (1993). Complete sequence of rabbit kidney aminopeptidase N and mRNA localization in rabbit kidney by in situ hybridization. Biochem Cell Biol.

[B78] Wakui H, Komatsuda A, Itoh H, Kobayashi R, Nakamoto Y, Miura AB (1992). Renal argininosuccinate synthetase: purification, immunohistochemical localization, and elastin-binding property. Ren Physiol Biochem.

[B79] Yanagita M, Oka M, Watabe T, Iguchi H, Niida A, Takahashi S, Akiyama T, Miyazono K, Yanagisawa M, Sakurai T (2004). USAG-1: a bone morphogenetic protein antagonist abundantly expressed in the kidney. Biochem Biophys Res Commun.

[B80] Dinjens WN, ten Kate J, Wijnen JT, van der Linden EP, Beek CJ, Lenders MH, Khan PM, Bosman FT (1989). Distribution of adenosine deaminase-complexing protein in murine tissues. J Biol Chem.

[B81] Fan CM, Kuwana E, Bulfone A, Fletcher CF, Copeland NG, Jenkins NA, Crews S, Martinez S, Puelles L, Rubenstein JL, Tessier-Lavigne M (1996). Expression patterns of two murine homologs of Drosophila single-minded suggest possible roles in embryonic patterning and in the pathogenesis of Down syndrome. Mol Cell Neurosci.

[B82] Leimeister C, Schumacher N, Gessler M (2003). Expression of Notch pathway genes in the embryonic mouse metanephros suggests a role in proximal tubule development. Gene Expr Patterns.

